# Effizienzsteigerung im patientenfernen Bereich bei delegierten Hausbesuchen in einer ländlichen Region Bayerns: Zwischenergebnisse der Studie VERSORGT am ORT

**DOI:** 10.1055/a-2620-5842

**Published:** 2025-07-08

**Authors:** Reiner Hofmann, Romina Lörzing, Martin Emmert, Julia Bräuer

**Affiliations:** 1 Medizincampus Oberfranken, Klinikum Bayreuth GmbH, Bayreuth, Germany; 2 Projektgeschäftsstelle Medizincampus Oberfranken, Universität Bayreuth, Bayreuth, Germany; 3 Institut für Medizinmanagement und Gesundheitswissenschaften, Universität Bayreuth, Bayreuth, Germany; 4 Bayerisches Krebsregister, Bayerisches Landesamt für Gesundheit und Lebensmittelsicherheit, Erlangen, Germany

**Keywords:** Allgemeinmedizin, Delegation, Effizienz, Hausbesuche, Ländliche, Gesundheitsversorgung, Primärversorgung, General practice, delegation, efficiency, home visits, rural healthcare, primary care

## Abstract

**Hintergrund und Ziel:**

Vor dem Hintergrund zunehmender Versorgungsprobleme in ländlichen Regionen ist die Weiterentwicklung ambulant-medizinischer Konzepte unter Fokus auf das Gebot der Wirtschaftlichkeit und Sparsamkeit unerlässlich. Die Delegation ärztlicher Aufgaben an medizinische Fachangestellte (MFA) bietet einen vielversprechenden Ansatz zur Entlastung von Hausärzt:innen und zur Unterstützung der Sicherstellung einer nachhaltigen Versorgung. Das Projekt "VERSORGT am ORT" (VaO) entwickelt dieses Prinzip weiter und zielt darauf ab, durch die partielle Verlagerung delegierter Hausbesuche in spezialisierte Versorgungsräume die patientenferne Arbeitszeit zu reduzieren, ohne die Versorgungsqualität zu beeinträchtigen.

**Methodik:**

Zur Evaluation des VaO-Konzepts werden als Parameter die Lebensqualität (LQ) und die Zufriedenheit (PZ) der Patient*innen über standardisierte Fragebögen sowie die für die Versorgung benötigte patientenferne Zeit und die Wegstrecken erhoben. Eine erste Datenauswertung von Mai 2024 bis Mai 2025 vergleicht den Standard in Form von Hausbesuchen in der Häuslichkeit (Hausbesuchs-/HB-Szenario) mit der teilweisen Verlagerung in VaO-Räume nach dem VaO-Modell (VaO-Szenario) mittels gepaarten t-Tests über zwei Erhebungszeitpunkte.

**Ergebnisse:**

Die analysierten n=134 Datensets zeigen im Szenarienvergleich signifikante Unterschiede zugunsten des VaO-Modells. Die Mittelwertdifferenzen liegen für die Fahrzeit bei MW=1,89 [min], Wegzeit bei MW=2,79 [min], Rüstzeit bei MW=0,63 [min], Gesamtzeit bei MW=5,32 [min] und für die gefahrenen Kilometer bei MW=1,89 [km] mit p-Werten<0.001. Insgesamt konnten eine Reduktion der patientenfernen Arbeitszeit um 20,9% von 25,32 auf 20,01 min und der gefahrenen Kilometer um 7,8% von 13,34 auf 12,30 km erreicht werden. Die LQ der Patient:innen verbesserte sich in der psychischen Komponente von MW=52,94 auf MW=57,27 signifikant. Die körperliche Summenskala stieg leicht von MW=41,01 auf MW=43,94. Die Zufriedenheit blieb nahezu unverändert.

**Schlussfolgerung:**

Diese Ergebnisse deuten darauf hin, dass die VaO-Systematik die Effizienz der hausärztlichen Versorgung in ländlichen Gebieten signifikant steigern kann. Trotz einer geringen absoluten Differenz der Zahlen zeigt der relative Vergleich bereits in dieser Größenordnung das Potential und die Effizienzsteigerung der innovativen Versorgungsform auf. Zudem zeigt die Zwischenevaluation, dass das Konzept nicht zu negativen Effekten für die LQ und die Zufriedenheit der Patient:innen führt. Eine Ausweitung auf weitere Regionen und eine tiefgreifende Analyse der Outcomeparameter durch weitere Studien werden empfohlen.

## Abstract (en)

**Background and objective:**

Against the background of increasing problems in the provision of medival care in rural regions, the further development of outpatient medical concepts focussing on the imperative of economic efficiency and economy is essential. The delegation of medical tasks to medical assistants (MFA) offers a promising approach to relieve the burden on GPs and to support the provision of sustainable care. The ‘VERSORGT am ORT’ (VaO) project develops this principle and aims to reduce working hours away from patients by partially shifting delegated home visits to specialised care rooms without compromising the quality of care.

**Methodology:**

Parameters for evaluating the VaO concept were quality of life (LQ) and satisfaction (PZ) of patients through standardized questionnaires, time spent away from patients and distance travelled. An initial data analysis from May 2024 to May 2025 compared the standard in the form of home visits (home visit /HB scenario) with partial transfer to VaO rooms according to the VaO model (VaO scenario) using paired t-tests over two survey time points.

**Results:**

The n=134 data sets analysed showed significant differences in favour of the VaO model in the scenario comparison. The mean differences for travel time were MW=1.89 [min], travel time MW=2.79 [min], set-up time MW=0.63 [min], total time MW=5.32 [min] and kilometres travelled MW=1.89 [km] with p-values<0.001. Overall, a 20.9% reduction in working time away from the patient from 25.32 to 20.01 min and a 7.8% reduction in kilometres travelled from 13.34 to 12.30 km were achieved. The psychological component of the patientsʼ LQ improved significantly from MW=52.94 to MW=57.27. The physical sum scale increased slightly from MW=41.01 to MW=43.94. Satisfaction remained almost unchanged.

**Conclusion:**

These results indicate that the VaO system can significantly increase the efficiency of GP care in rural areas. Despite a small absolute difference in the figures, the relative comparison already shows the potential and the increased efficiency of this innovative form of care. In addition, the interim evaluation shows that the concept does not lead to negative effects on QoL and patient satisfaction. An expansion to other regions and an in-depth analysis of the outcome parameters in further studies are recommended.

## Einleitung

Vor dem Hintergrund der Versorgungsprobleme ländlicher Regionen steigt der Bedarf an innovativen ambulanten Versorgungskonzepten. Zur Zukunftssicherung der Versorgung erweist sich die Delegation ärztlicher Aufgaben an qualifizierte medizinische Fachangestellte (MFA) als zielführend 1 . Dies entlastet Hausärzt*innen in der Praxis wie auch bei Routine-Hausbesuchen (HB) 2 . Die Umverteilung von Aufgaben durch Delegation kann aus Sicht der Hausärzt*innen personelle Ressourcen effizienter einsetzen und sich vorteilhaft auf die Hausarztpraxis, deren Patient*innen sowie die Versorgung generell auswirken 3 .

Bei delegierten Routine-HB werden alle Patient*innen einzeln zu Hause besucht und versorgt. Geeignete HB werden zu Touren zusammengefasst. Das Konzept VERSORGT am ORT (VaO) zielt darauf ab, die HB-Touren effizienter zu gestalten, ohne die Versorgungsqualität negativ zu beeinflussen. Nach ärztlicher Feststellung der Eignung und informierter Zustimmung der Patient*innen werden delegierte HB in speziell eingerichtete Versorgungsräume verlagert. Der Leistungsumfang und die leistungserbringenden MFA bleiben dabei unverändert, einzig der Ort der Leistungserbringung wird verändert. In einer Versuchsregion wird anhand eines kontrollierten Studien-Settings untersucht, ob sich Wegstrecken und Arbeitszeiten im patientenfernen Bereich reduzieren sowie die gesundheitsbezogene Lebensqualität (LQ) und Zufriedenheit der Patient*innen (PZ) auf einem zumindest konstanten Niveau bleiben.

## Methodik

*Datenerhebung* Zur Evaluation des innovativen Versorgungskonzepts VaO werden (1) die gefahrenen Kilometer und (2) die benötigte Zeit der MFA sowie die (3) LQ und (4) die PZ der Patient*innen erhoben. Ein positives Votum der Ethikkommission der Universität Bayreuth (Vorgangsnummer 23-014) liegt vor.

Die vorliegende Zwischenauswertung zeigt die Ergebnisse für die Parameter Zeit und Wegstrecke basierend auf von Mai 2023 bis Mai 2024 durch drei teilnehmende Praxen in acht VaO-Räumen erhobenen Daten. Die Wegstrecken beziehen sich auf die Entfernungen, die von den MFA in ihren HB-Touren zurückgelegt werden. Mithilfe des Routenplaners MapQuest werden die kürzesten Routen in zwei Szenarien berechnet: Das HB-Szenario umfasst als üblicher Standard die Adressen aller Patient*innen, welche durch delegierte HB versorgt werden. Die Wegstreckenberechnung im VaO-Szenario umfasst die Adressen der VaO-Räume und der Patient*innen, welche nicht im VaO-Raum versorgt werden. Dabei werden sowohl die zurückgelegten Kilometer (km) als auch die benötigte Zeit (min) ermittelt. Der Outcome-Parameter Zeit umfasst die zwei Teilgrößen Wegstreckenzeit (Fahrt- und Fußwege) sowie die Setup-Zeit zur Vor- und Nachbereitung der medizinischen Leistung. Die mittleren Setup-Zeiten wurden vorab in einem Workshop mit den teilnehmenden MFA gemessen. Inhalt und Dauer der Patientenversorgung bleiben unverändert und werden nicht in die Auswertung einbezogen.

Zur Erfassung der LQ wird der Fragebogen Short Form 12 (SF-12) mit den Subskalen körperliche und psychische Summenskala (KSK, PSK, Min-Max-Werte jeweils 0-100) verwendet 4 . Die PZ wird mittels des Fragebogens ZUF-8 (Min-Max-Werte 0-32) dokumentiert 5 . Die Erhebung dieser beiden Parameter erfolgt zu drei Zeitpunkten aus individueller Sicht der fortlaufend eingeschlossenen Patient*innen im VaO-Szenario: vor Beginn der Versorgung im VaO-Raum zur Erfassung der Basisdaten (t0), nach sechs Monaten (t1) und nach weiteren zwölf Monaten (t2). *Datenauswertung* Die Auswertung erfolgte mit dem Statistikprogramm IBM Corp. Released 2023. IBM SPSS Statistics for Windows, Version 29.0.2.0 Armonk, NY: IBM Corp. Um Unterschiede zwischen den Szenarien zu analysieren, wurden gepaarte t-Tests mit den Variablen gefahrene km und benötigte Zeit durchgeführt. Das Signifikanzniveau wurde mit alpha=0,05 festgelegt. Die aus den Fragebögen erhobenen Daten zur PZ und LQ wurden ebenfalls mit Hilfe von gepaarten t-Tests ausgewertet (alpha=0,05).

## Ergebnisse

Das durchschnittliche Alter der Studienteilnehmer*innen (n=110) beträgt 77,81 Jahre (SD=9,37). Die Analyse basiert auf n=134 bereinigten und vollständigen Szenarien-Datensets, die Berechnung der Zeiten umfasst 659 Leistungen (davon 196 in der Häuslichkeit und 463 bzw. 70% in den VaO-Räumen) aus 488 Patientenkontakten (222 in der Häuslichkeit, 266 bzw. 55% in den VaO-Räumen).

Die Mittelwertdifferenzen liegen bei 1,04 km und 5,31 min, einer Reduzierung um 7,8% der Wegstrecken und 20,9% der patientenfernen Arbeitszeit entsprechend. Die gefahrenen Wegstrecken sind im VaO-Szenario mit 12,30 km (SD=6,09) im Mittel geringer als im HB-Szenario mit 13,34 km (SD=6,47). Die Effektgröße Cohen’s d liegt bei 1,59. Daneben untergliedert sich die Wegstreckenzeit in Fahrtzeit mit dem Auto und Fußwegzeit vom Stellplatz zum Ort der Leistungserbringung. Im VaO-Szenario beträgt die mittlere Fahrtzeit 13,93 min (SD=5,63), während im HB-Szenario 15,82 min (SD=5,83) gemessen werden (d=1,76). Die durchschnittliche Fußwegzeit ist im VaO-Szenario mit 4,49 min (SD=3,33) kürzer als im HB-Szenario mit 7,28 min (SD=4,38, d=2,61). Auch die Setup-Zeit ist im VaO-Szenario mit 1,59 min (SD=1,18) kürzer als im HB-Szenario mit 2,22 min (SD=1,50, d=0,89). Alle Paare weisen statistisch signifikante Unterschiede mit p<0,001 auf (s. Tab. 1 ).

**Table:** **Tab. 1** Statistische Auswertung

	VaO (MW)	HB (MW)	Δ	Δ in %	95% KI	Einseitiges p	Cohen’s d
Strecken [km]	12,3	13,34	1,04	7,80%	[0,76;1,31]	<0,001	1,59
Fahrtzeit [min]	13,93	15,82	1,9	12,00%	[1,59;2,19]	<0,001	1,76
Fußweg [min]	4,49	7,28	2,79	38,30%	[2,35;3,24]	<0,001	2,61
Setup [min]	1,59	2,22	0,63	28,40%	[0,47;0,78]	<0,001	0,89
Gesamtzeit [min]	20,01	25,32	5,31	20,90%	[4,56;6,06]	<0,001	4,39
	**T0 (MW)**	**T1 (MW)**	**Δ**	**Δ in %**	**95% KI**	**Zweiseitiges p**	**Cohen’s d**
Lebensqualität KSK (n=48)	41,01	43,94	2,93	7,14%	[− 5,95;0,1]	0,057	− 0,28
Lebensqualität PSK (n=48)	52,94	57,27	4,33	8,18%	[− 7,55;-1,1]	0,01	− 0,39
Zufriedenheit (n=72)	30,49	30,35	0,14	0,46%	[− 0,26;0,54]	0,49	0,08

Die durchschnittliche PZ (n=72) beträgt zum Messzeitpunkt t0 30,49 (SD=2,31) und zum Messzeitpunkt t1 30,37 (SD=2,30). Der Unterschied ist nicht signifikant (p=0,49, d=0,08). Bei der Betrachtung der LQ (n=48) ergibt sich zum Zeitpunkt t0 Mittelwerte für die KSK von 41,01 (SD=11,88), zu t1 von 43,94 (SD=11,07). Für die PSK werden Mittelwerte von 52,94 (SD=9,5) zu t0 und von 57,27 (SD=5,38) für t1 ermittelt. Hier zeigt sich ein statistisch signifikanter Unterschied bei der PSK (p=0,01, d=− 0,39) im Gegensatz zur KSK mit p=0,057 (d=− 0,28) (s. Tab. 1 ).

## Diskussion

Die vorliegenden Ergebnisse der ersten Evaluation zeigen, dass VaO die Wegstrecken und patientenferne Arbeitszeit reduziert, ohne zu negativen Effekten für die LQ und PZ zu führen. Die kleine Gruppe der nach den regulatorischen sowie im Studienprotokoll festgelegten Ein- und Ausschlusskriterien ausgewählten VaO-geeigneten Patient*innen wird einerseits durch die medizinische Notwendigkeit eines HB und andererseits durch die Mobilität zum Besuch der VaO-Räume eingegrenzt und verändert sich fortlaufend. Daraus resultieren abweichende Touren, Strecken und Zeiten. Alle getesteten Parameter weisen statistisch signifikante Differenzen (p<0,001) und hohe Effektstärken (Cohen’s d>0,8) auf. Dies weist auf sehr ausgeprägte Unterschiede hin. Trotz einer geringen absoluten Differenz der Zahlen zeigt der relative Vergleich bereits in dieser Größenordnung das Potential und die Effizienzsteigerung der innovativen Versorgungsform. Zur noch ausstehenden Analyse der Generalisierbarkeit des VaO-Konzepts wird dieses im weiteren Projektverlauf in einer Vergleichsregion implementiert und evaluiert. Trotz der Vorteilhaftigkeit sind weitere Analysen erforderlich, um beispielsweise regional bedingte interne wie externe Faktoren oder infrastrukturelle Unterschiede tiefergehend zu untersuchen.

Die hohen t0-Werte der PZ weisen darauf hin, dass die Patient*innen mit der bisherigen hausärztlichen Versorgung über HB in der Untersuchungsregion zufrieden sind. Die im Vergleich unveränderten t1-Werte zeichnen ein positives Bild der innovativen Versorgungsform, wobei auch diese Datenbasis im weiteren Projektverlauf durch neu in das VaO-Konzept aufgenommene Patient*innen fortlaufend erweitert wird, was den Nachteil des geringen Stichprobenumfangs verringert.

Die Ergebnisse der LQ-Erhebungen lassen sich in vergleichbare Stichproben einordnen. Im Vergleich zur Normstichprobe des SF-12 in der entsprechenden Altersgruppe liegen die t0-Werte der VaO-Population mit KSK=41,01 und PSK=52,94 über den Norm-MW der beiden Subskalen (KSK=38,76 und PSK=52,27) oder den von Schmidt und Gebert ermittelten MW KSK=39,67 und PSK=48,69 6 . Dies unterstreicht die Robustheit der erhobenen Daten. In t1 ergeben sich MW von KSK=43,94 und PSK=52,27, was nahelegt, dass VaO insbesondere auf die psychische Gesundheit einen positiven Einfluss hat, wohingegen bei der körperlichen Lebensqualität bisher keine signifikante Veränderung feststellbar war. Zur Erörterung dieser Wirkung von VaO auf die beiden Subskalen sind weitere Untersuchungen durchzuführen, welche sich vor allem mit der Verbesserung der KSK auseinandersetzen. Insgesamt unterstützen die Ergebnisse die Annahme, dass VaO keinen negativen Einfluss auf die LQ der Patient*innen aufweist.

## Fördermittel

Bayerisches Staatsministerium für Gesundheit, Pflege und Prävention — G31f-G8000-2020/989-33

## Notes

Fazit Die Durchführung delegierter HB in speziell eingerichteten Räumen nach dem Konzept VaO führt zu einer signifikanten Effizienzsteigerung in der patientenfernen Arbeitszeit, ohne LQ und PZ zu beschneiden. Die erzielte Zeitersparnis von 20,9% und die Wegstreckenverkürzung um 7,8% unterstreichen das Potenzial des weiterentwickelten Delegationsmodells, die Effizienz der hausärztlichen Versorgung erheblich zu steigern – ohne die Kombination aus Mediziner*in-MFA-Patient*in oder die erbrachte Leistung zu ändern, was sich auch in den Ergebnissen zur PZ sowie zur LQ widerspiegelt, die ein konstant hohes bis steigendes Niveau aufweisen. Um die Umsetzung und die Wirksamkeit dieses innovativen Modells zu verstetigen, bedarf es einer Anpassung der regulatorischen Rahmenbedingungen. Es ist entscheidend, dass zukünftige Strategien diesen zentralen Aspekt berücksichtigen, um eine flächendeckende Gesundheitsversorgung aufrechtzuhalten.
